# Palmatine Alleviates Particulate Matter-Induced Acute Lung Injury by Inhibiting Pyroptosis via Activating the Nrf2-Related Pathway

**DOI:** 10.1007/s10753-024-02009-2

**Published:** 2024-04-10

**Authors:** Hao Zuo, Wanting Zhou, Yijing Chen, Binqian Zhou, Zhengkai Wang, Shuai Huang, Tahereh Alinejad, Chengshui Chen

**Affiliations:** 1 Key Laboratory of Interventional Pulmonology of Zhejiang Province, The First Affiliated Hospital of Wenzhou Medical University, Wenzhou, 325015 China; 2 Department of Pulmonary and Critical Care Medicine, the First Affiliated Hospital of Wenzhou Medical University, Wenzhou, 325000 China; 3 Department of Pulmonary and Critical Care Medicine, the, Quzhou Affiliated Hospital of Wenzhou Medical University, Quzhou People’s Hospital, Quzhou, 324000 China; 4 Oujiang Laboratory (Zhejiang Lab for Regenerative Medicine, Institute of Cell Growth Factor, and Brain Health), Wenzhou Medical University, VisionWenzhou, China

**Keywords:** Palmatine, particulate matter, a cute lung injury, pyroptosis, Nrf2

## Abstract

Particulate matter (PM) induces and enhances oxidative stress and inflammation, leading to a variety of respiratory diseases, including acute lung injury. Exploring new treatments for PM-induced lung injury has long been of interest to researchers. Palmatine (PAL) is a natural extract derived from plants that has been reported in many studies to alleviate inflammatory diseases. Our study was designed to explore whether PAL can alleviate acute lung injury caused by PM. The acute lung injury model was established by instilling PM (4 mg/kg) into the airway of mice, and PAL (50 mg/kg and 100 m/kg) was administrated orally as the treatment groups. The effect and mechanism of PAL treatment were examined by immunofluorescence, immunohistochemistry, Western Blotting, ELISA, and other experiments. The results showed that oral administration of PAL (50 mg/kg and 100 m/kg) could significantly alleviate lung inflammation and acute lung injury caused by PM. In terms of mechanism, we found that PAL (50 mg/kg) exerts anti-inflammatory and anti-damage effects mainly by enhancing the activation of the Nrf2-related antioxidant pathway and inhibiting the activation of the NLRP3-related pyroptosis pathway in mice. These mechanisms have also been verified in our cell experiments. Further cell experiments showed that PAL may reduce intracellular reactive oxygen species (ROS) by activating Nrf2-related pathways, thereby inhibiting the activation of NLRP3-related pyroptosis pathway induced by PM in Beas-2B cell. Our study suggests that PAL can be a new option for PM-induced acute lung injury.

## Introduction

Exposure to ambient particulate matter (PM) is a significant risk factor for pulmonary inflammatory disease [1]. From 1990 to 2019, fine particulate matter (aerodynamic diameter <  = 2.5 μm, PM2.5) contributed to a staggering increase of 102.3% in global mortality rates [2]. PM is a complex mixture of particles and toxic substances emitted from the combustion of oil and fossil fuels [3]. It has the ability to deeply penetrate into the bronchioles and alveoli of the lungs, triggering an acute inflammatory response. Previous studies have shown that PM exposure can activate various signaling pathways associated with inflammation and injury in the respiratory, cardiovascular, and nervous systems, including NOD-like receptor thermal protein domain-associated protein 3 (NLRP3)-mediated pyroptosis and Nuclear factor erythroid 2-related factor 2 (Nrf2)-mediated oxidative stress pathways [4, 5]. The identification of novel approaches to inhibit inflammatory signals, mitigate disease progression, and reduce PM-induced damage holds paramount importance. Therefore, it is crucial to explore new therapeutic targets for drug development aimed at treating acute lung injury caused by PM exposure.

The Nrf2-related antioxidant pathway and the NLRP3-related pyroptosis play important roles in the pathogenesis of acute lung injury. The Nrf2 protein is sequestered by the Keap1 protein under normal conditions. However, when an excessive amount of reactive oxygen species (ROS) is generated within the cell, the Nrf2 protein dissociates from Keap1 and translocates into the nucleus, where it acts as a transcription factor to initiate the expression of oxidative stress-related genes such as HO-1 and NQO1 [6]. On the other hand, activation of the NLRP3 inflammasome occurs in response to inflammation or elevated ROS levels [7, 8]. Upon activation, NLRP3 recruits ASC and pro-Caspase-1 leading to its aggregation and subsequent self-proteolysis to generate enzymatically active Caspase-1. Caspase-1 then cleaves pro-IL-1β into IL-1β and gasdermin-D into its active form (N-terminal gasdermin-D), which forms membrane pores resulting in pyroptotic cell death [9]. Therefore, modulating these pathways by activating antioxidant responses while inhibiting inflammatory pyroptosis represents promising strategies for mitigating acute lung injury.

Palmatine (PAL) is a naturally occurring isoquinoline alkaloid found in many traditional Chinese medicines [10–12]. Extensive research have been conducted on the pharmacological effects of PAL, encompassing neuroprotection, anti-cancer properties, antibacterial and antiviral activities, antioxidant and anti-inflammatory effects, as well as regulation of blood lipids [13]. These studies have demonstrated the significant potential of PAL in preventing and treating several diseases such as cancer, heart hypertrophy, osteoporosis, diabetes and its complications, Alzheimer’s disease, atopic dermatitis, and osteoarthritis [14–16]. However, there is currently no available literature investigating whether PAL can alleviate PM-induced acute lung injury. Therefore, this study aims to investigate both the protective effect and underlying mechanism of PAL against PM-induced acute lung injury *in vivo* and *in vitro*.

## Materials and Methods

### Reagents and Antibodies

PM (The standard reference airborne 1649b) was purchased from the National Institute of Standards and Technology (NIST, Gaithersburg, USA). PAL (powder) was obtained from Macklin (Shanghai, China). TUNEL detection kit and ROS detection kit were purchased from Beyotime (Shanghai, China). The ELISA kits of IL-18, IL-6, IL-8, SOD, MDA, and IL-1β were purchased from Boyun Biotechnology (Shanghai, China). The DMEM/F12 medium, PBS, and penicillin–streptomycin were purchased from Gibco (USA), and the fetal bovine serum (FBS) was purchased from Sijiqing (Hangzhou, China). RIPA lysis buffer and phenylmethylsulfonyl fluoride (PMSF) were purchased from Solarbio (Beijing, China) while the phosphatase inhibitors were purchased from Applygen (Beijing, China). Antibodies against Nrf2, ASC, IL-18, IL-1β, Cleaved-caspase-1, and β-actin were purchased from Abclonal (Wuhan, China); antibodies against NLRP3, HO-1, NQO1, and N-GSDMD were purchased from Affinity (Jiangsu, China); antibody against EpCAM was purchased from Santa (Cruz, USA); the secondary antibody for Western Blotting was purchased from Biosharp (Beijing, China); ML385 was purchased from MedChemExpress (Shanghai, China).

### Cell Culture and Intervention

Frozen human normal lung epithelial cells Beas-2B (ATCC, USA) were removed from the liquid nitrogen tank, thawed in water at 37 ℃ until the frozen solution dissolved, and the cell suspension was transferred to a centrifuge tube and centrifuged at 1000 rpm for 3 min. Remove the supernatant, add 1 mL DMEM/F12 medium to resuspend cells, then transfer cells to culture bottle, add 4 mL DMEM/F12 medium containing 10% FBS and 1% penicillin/streptomycin, and culture in 37 ℃, 5% CO_2_ incubator. When the cell confluence reached 80%, the culture was subcultured to 6-well plate.

We have four groups in the cellular experiment, including the control group, PAL group, PM group, and PM + PAL group. After the confluence of cell in the 6-well plate reached 80%, the PM group and PM + PAL group were administrated with PM first; 2 h later, we gave PAL to the PM + PAL group. According to previous literature, we set concentrations of PM and PAL as 200 μg/ml and 80 μM, respectively [17, 18]. After 24 h, cells were collected for the next step.

For the cellular experiments that explored the mechanisms, we also set four groups, including the control group, PM group, PM + PAL group, and PM + PAL + ML385 group. ML385 is a specific Nrf2 inhibitor, and the dosage we give was according to previous literature [19]. After the confluence of cell in the 6-well plate reached 80%, the PM group, PM + PAL group, and PM + PAL + ML385 group were administrated with PM (200 μg/ml) first, and we gave PAL (80 μM) with or without ML385 (10 μM) to the PM + PAL group and PM + PAL + ML385 group 2 h later. After 24 h, the cells were collected for the next step.

### Animals Experiment

Animal experiments were conducted using specific pathogen-free (SPF) grade C57BL/6 J male mice, aged 6–8 weeks and weighed 20–22 g, purchased from Vital River Laboratory Animal Technology Company. All animal experiments were approved by the Animal Care and Use Committee of the First Hospital of Wenzhou Medical University. After 7 days of adaptation, the mice were randomly divided into five groups: (I) control group, (II) PAL group, (III) PM group, (IV) PM + PAL (50 mg/kg) group, and (V) PM + PAL (100 mg/kg) group. According to the previous literature, we set the dosage of PM to be administered to mice at 4 mg/kg [20]. PM and PAL were both dissolved in saline. On the first day, mice were administrated orally with saline or PAL (200 μL) 2 h after intratracheal instillation with saline or PM (50 μL). On the second day, the same operation was applied to the mice. The mice were humanely euthanized 48 h after the first PM administration. Lung tissue and bronchoalveolar lavage fluid (BALF) were then collected.

### Western Blotting

Proteins of Beas-2B cells and lung tissue were extracted using RIPA lysis buffer, followed by SDS–polyacrylamide gel electrophoresis, and then transferred to nitrocellulose membranes. After incubation with primary antibody, the membranes were performed with appropriate secondary antibody. The protein bands were observed by enhanced chemiluminescence. At least three independent immunoblots were used to provide quantitative protein analysis. The gray values of protein bands were quantified by the ImageJ software.

### HE Staining

Lung tissues were collected for histopathological examination immediately after euthanization. The lung specimens were fixed with 4% paraformaldehyde at room temperature for 24 h and then dehydrated and embedded. After dewaxing and dehydrating, the lung sections were cut to 5 μm. These lung sections were stained with hematoxylin and eosin (H&E) to assess histopathological changes. The pathological changes in the lung tissues were observed with a light microscope and evaluated on a lung injury score of 1 to 4. The pathological criteria were as follows: (1) no inflammation; (2) occasional cuffing with inflammatory cells; (3) most bronchi or vessels surrounded by a thin layer (1 to 5) of inflammatory cells; (4) most bronchi or vessels surrounded by a thick layer (> 5) of inflammatory cells [20].

### TUNEL Staining

The lung specimens were fixed with 4% paraformaldehyde at room temperature for 24 h. After dewaxing and dehydrating, the lung sections were cut to 5 μm. Subsequently, the lung tissue sections were stained according to the instructions. TUNEL staining of cells was also performed according to the instructions. The proportion of TUNEL-positive cells was quantified by ImageJ software.

### ROS Detection

Twenty-four hours after the cells were given the intervention, cells were washed by PBS and then incubated with DCFH-DA solution (10 µM) at 37 °C in the dark for 30 min and hoechst for 10 min. Photographs were taken with a fluorescence microscope within 30 min (excitation wavelength was 488 nm, emission wavelength was 525 nm). The mean fluorescence intensity of ROS was quantified by ImageJ software.

### Immunofluorescence Staining

Lung tissues were fixed, paraffin-embedded, and sectioned. After treatment with antigen retrieval, lung sections were incubated with designated antibodies in a 4 ℃ refrigerator overnight, followed by incubation with corresponding IgG secondary antibodies, and finally stained with DAPI solution. The positive rates of NLRP3 and Nrf2 in airway epitheliums were quantified by using ImageJ software to calculate the ratio of the number of NLRP3 or Nrf2 positive cells to the EpCAM positive cells in the airway.

### Statistical Analysis

All data are expressed as the mean ± standard deviation (SD) of at least three independent experiments. Student’s *t*-test was used for comparison between different groups. For multiple comparisons, one-way analysis of variance (ANOVA) was used. Statistical analysis was performed using Prism 8 (GraphPad Prism, version 8.0.1). *P* < 0.05 was considered statistically significant.

## Result

### PAL Significantly Ameliorate PM-Induced Airway Epithelial Injury

**Fig. 1:**
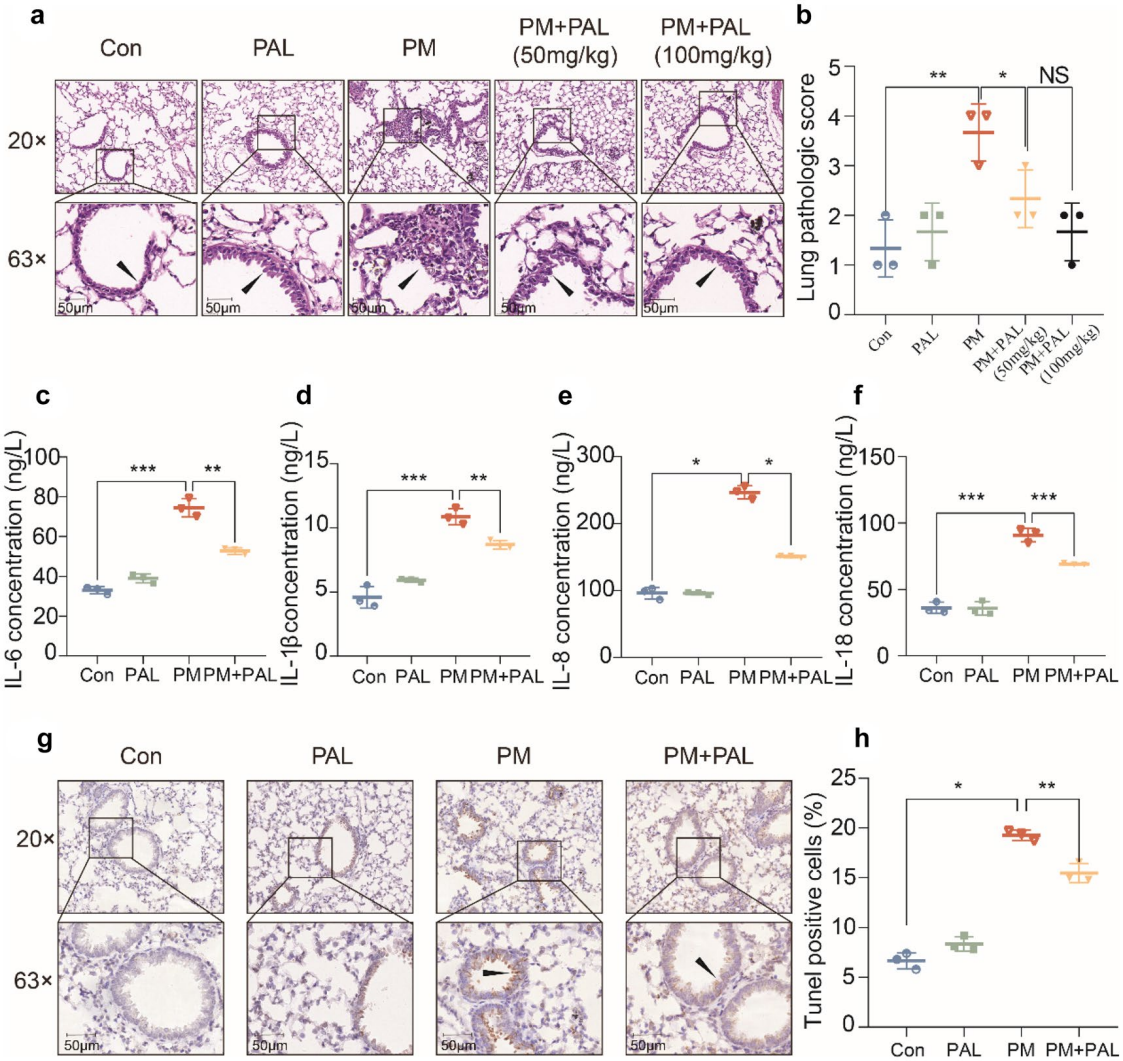
PAL alleviates acute lung injury and inflammation induced by PM. **a** Representative H&E staining of sections of lung tissues of mice in different groups. The black arrows indicate the airway epithelium and surrounding inflammatory cells that we are interested in. **b** Lung pathologic scores of mice in different groups. **c** IL-6 concentration of BALF in each group (*n* = 3). **d** IL-1β concentration of BALF in each group (*n* = 3). **e** IL-8 concentration of BALF in each group (*n* = 3). **f** IL-18 concentration of BALF in each group (*n* = 3). **g** Representative TUNEL staining of sections of lung tissues of mice in different groups. The black arrows indicate the apoptotic cells that we focus on. **h** Percentage of TUNEL positive cell number relative to the total number of nuclei (*n* = 3). *p < 0.05; **p < 0.01, ***p < 0.001. One-way ANOVA was used for multiple comparisons.

H&E staining was used to detect the degree of lung injury in mice, and the results showed that PAL (50 mg/kg and 100 mg/kg) could significantly ameliorate airway epithelial injury caused by PM (Fig. 1a, b). We found that there was no significant difference in the therapeutic effect between PAL administration of 50 mg/kg and 100 mg/kg on acute lung injury in mice, so the results of subsequent animal experiments were all from mice in the PAL intervention group of 50 mg/kg. Results of ELISA showed that PAL (50 mg/kg) treatment significantly reduced PM-induced inflammatory cytokine concentrations of BALF (Fig. 1c–f). The apoptosis of airway epithelium was further detected by TUNEL staining, and the results showed that PAL (50 mg/kg) could significantly reduce the apoptosis of airway epithelium cells (Fig. 1g, h).

### PAL Significantly Inhibited the Activation of NLRP3-Related Pyroptosis Pathway Induced by PM in Lung of Mice

**Fig. 2:**
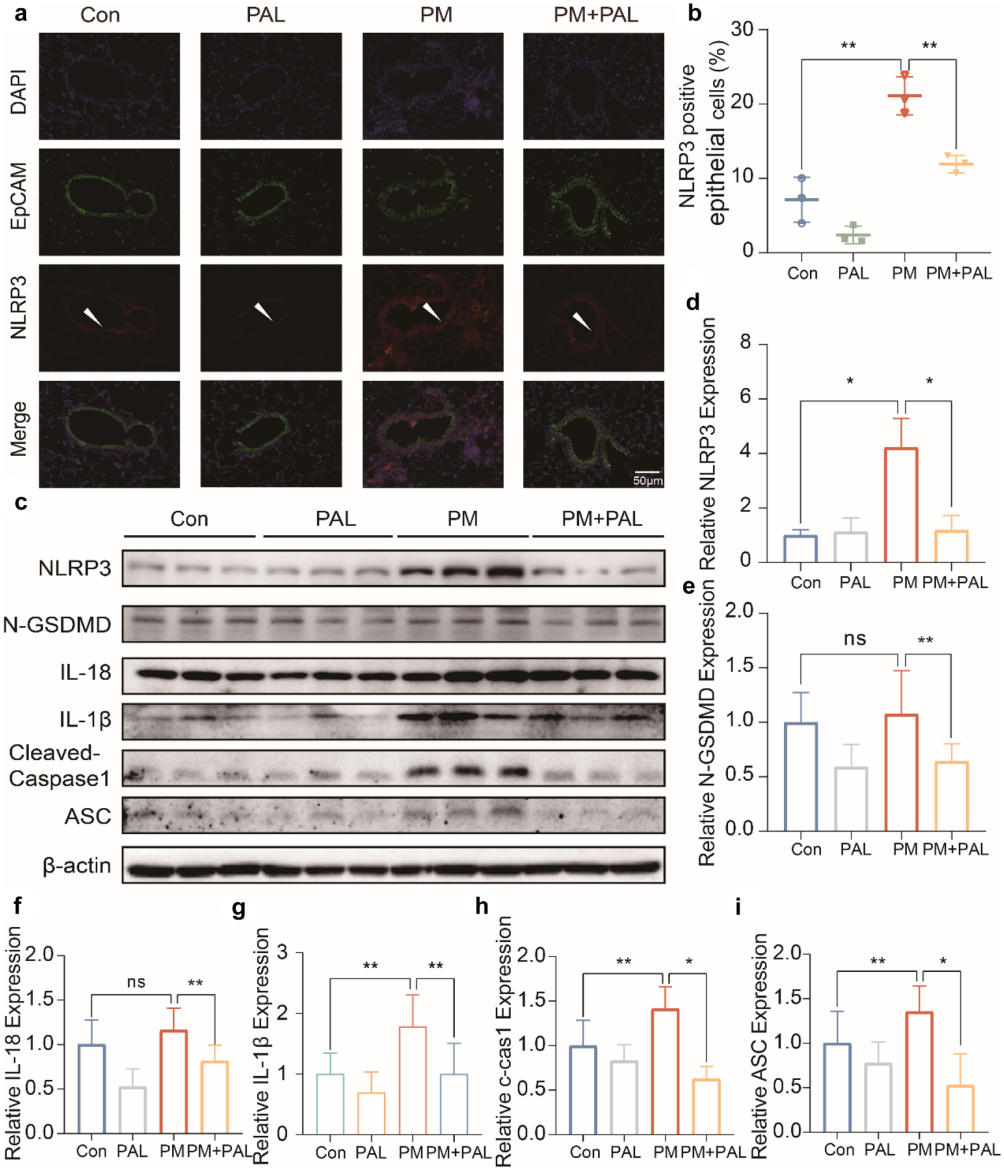
PAL alleviates acute lung injury in mice by inhibiting activation of NLRP3-related pyroptosis pathway. **a** Representative IF staining of NLRP3 and EpCAM protein of lung tissues of mice in different groups. The white arrows indicate the airway epithelium (EpCAM positive) that we are interested in. **b** Percentage of NLRP3 and EpCAM double positive cell number relative to the total number of EpCAM positive cell numbers (*n* = 3). **c** Representative protein bands of NLRP3-related pathways by Western Blotting of lung tissue protein of mice. **d** Western Blotting analysis of NLRP3 protein (*n* = 3). **e** Western Blotting analysis of N-GSDMD protein (*n* = 3). **f** Western Blotting analysis of IL-18 protein (*n* = 3). **g** Western Blotting analysis of IL-1β protein. **h** Western Blotting analysis of Cleaved-Caspase3 protein (*n* = 3). **i** Western Blotting analysis of ASC protein (*n* = 3). *p < 0.05; **p < 0.01, ***p < 0.001. One-way ANOVA was used for multiple comparisons.

In order to further explore the effect of PAL (50 mg/kg) on the NLRP3-related pyroptosis pathway, we co-stained EpCAM and NLRP3 by immunofluorescence, and it was found that NLRP3 protein expression was significantly increased in the airway epitheliums of mice after PM administration, while PAL (50 mg/kg) inhibited the up-regulation of NLRP3 protein (Fig. 2a, b). The lung tissue protein of mice was extracted for Western Blotting. The results showed that the protein expression of the NLRP3-related pyroptosis pathway were significantly increased in the lung tissue of mice treated with PM, while PAL (50 mg/kg) could inhibit the activation of this pathway (Fig. 2c–i).

### PAL Significantly Activated Nrf2-Related Antioxidant Pathway in Lung of Mice

**Fig. 3:**
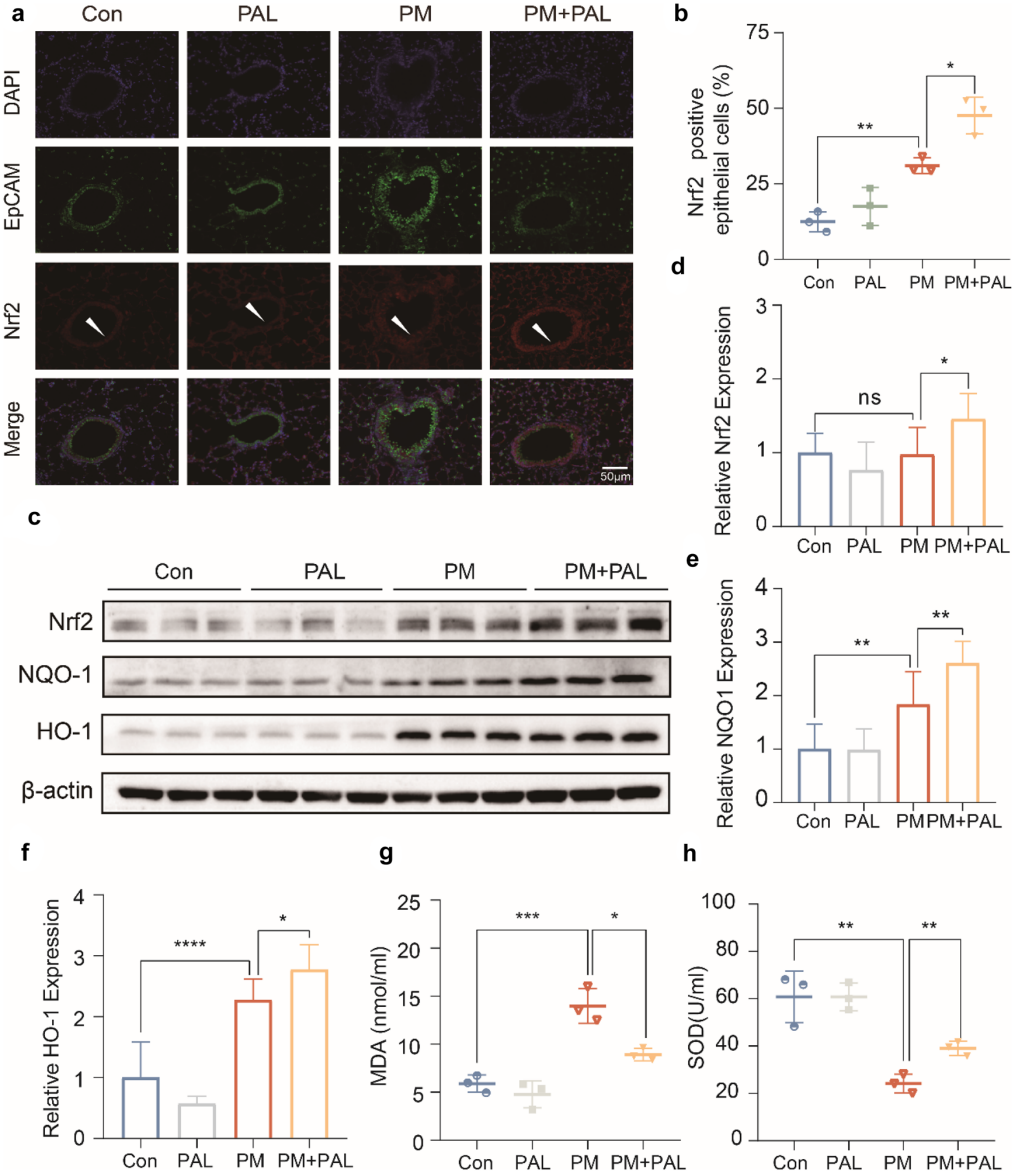
PAL alleviates acute lung injury in mice by enhancing the activation of Nrf2-related pathway. **a** Representative IF staining of Nrf2 and EpCAM protein of lung tissues of mice in different groups. The white arrows indicate the airway epithelium (EpCAM positive) that we are interested in. **b** Percentage of Nrf2 and EpCAM double positive cell number relative to the total number of EpCAM positive cell numbers (*n* = 3). **c** Representative protein bands of Nrf2-related pathways by Western Blotting of lung tissue protein of mice. **d** Western Blotting analysis of Nrf2 protein (*n* = 3). **e** Western Blotting analysis of NQO1 protein (*n* = 3). **f** Western Blotting analysis of HO-1 protein (*n* = 3). **g** MDA concentration of BALF in each group (*n* = 3). **h** SOD activity of BALF in each group (*n* = 3). *p < 0.05; **p < 0.01, ***p < 0.001. One-way ANOVA was used for multiple comparisons.

ROS plays an important role in acute inflammation, and the activation of the Nrf2-related pathways can clear ROS and protect cells and tissues from the damage caused by ROS. We co-stained EpCAM and Nrf2 proteins by immunofluorescence and found that Nrf2 protein expression was significantly up-regulated in airway epithelium after PM administration, and PAL (50 mg/kg) could enhance the up-regulation of Nrf2 protein (Fig. 3a, b). The lung tissue protein of mice was extracted for Western Blotting. The results of Western Blotting showed that PM administration significantly activated Nrf2-related pathways, and PAL (50 mg/kg) could further enhance the activation of Nrf2-related pathways (Fig. 3c–f). Results of ELISA showed that PAL (50 mg/kg) treatment significantly reduced MDA concentration and increased SOD activity of BALF in the PM + PAL group (Fig. 3g, h).

### PAL Significantly Inhibited the Activation of NLRP3-Related Pyroptosis Pathway Induced by PM in Beas-2B Cell Line

**Fig. 4:**
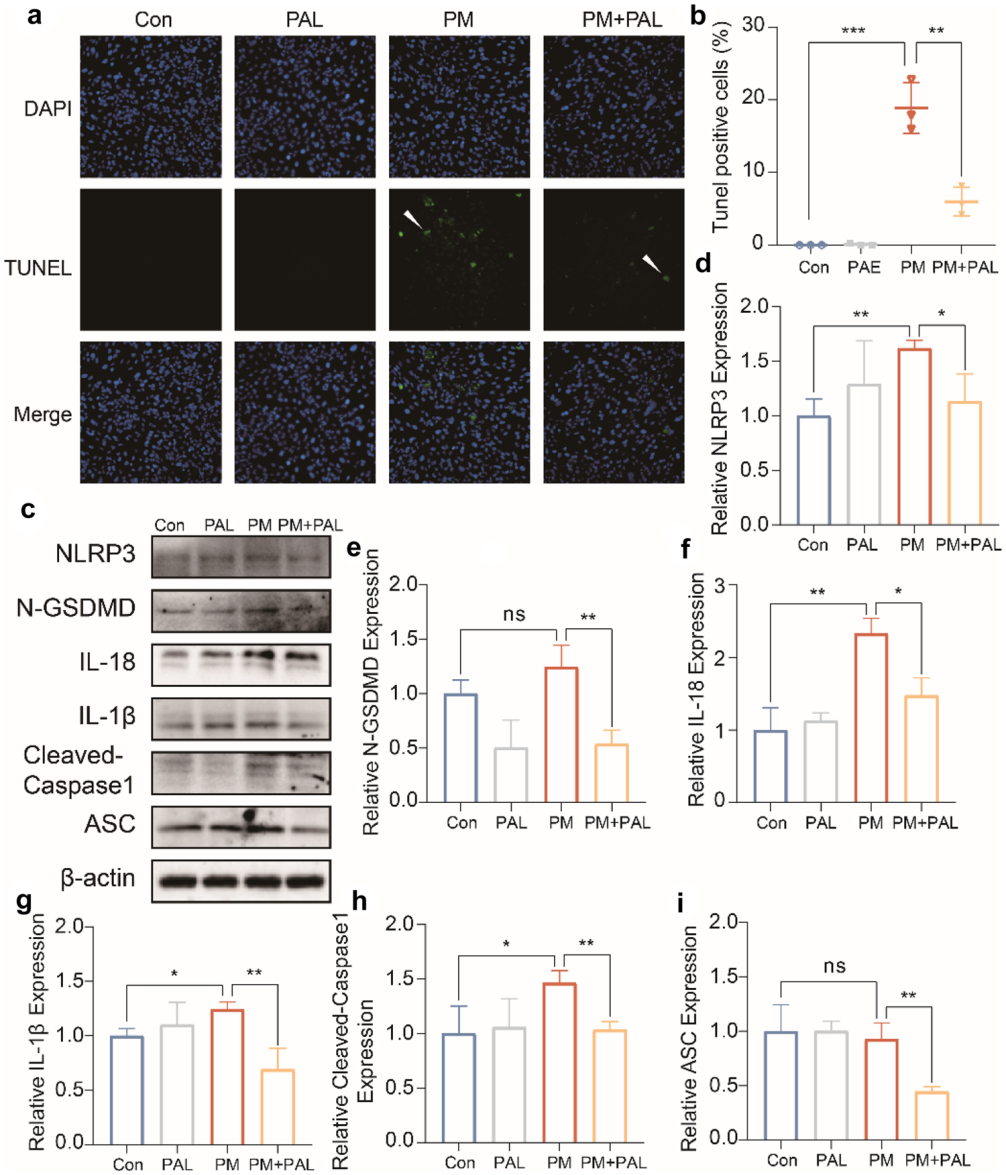
PAL alleviates the damage of Beas-2B cells induced by PM administration by inhibiting the NLRP3-associated pyroptosis pathway. **a** Representative IF staining of TUNEL staining of Beas-2B cells in different groups. The white arrows indicate the apoptotic cells that we focus on. **b** Percentage of TUNEL positive cell number relative to the total number of Hoechst (*n* = 3). **c** Representative protein bands of NLRP3-related pathways by Western Blotting of Beas-2B cell in different groups. **d** Western Blotting analysis of NLRP3 protein (*n* = 3). **e** Western Blotting analysis of N-GSDMD protein (*n* = 3). **f** Western Blotting analysis of IL-18 protein (*n* = 3). **g** Western Blotting analysis of IL-1β protein. **h** Western Blotting analysis of Cleaved-Caspase3 protein (*n* = 3). **i** Western Blotting analysis of ASC protein (*n* = 3). *p < 0.05; **p < 0.01, ***p < 0.001. One-way ANOVA was used for multiple comparisons.

**Fig. 5:**
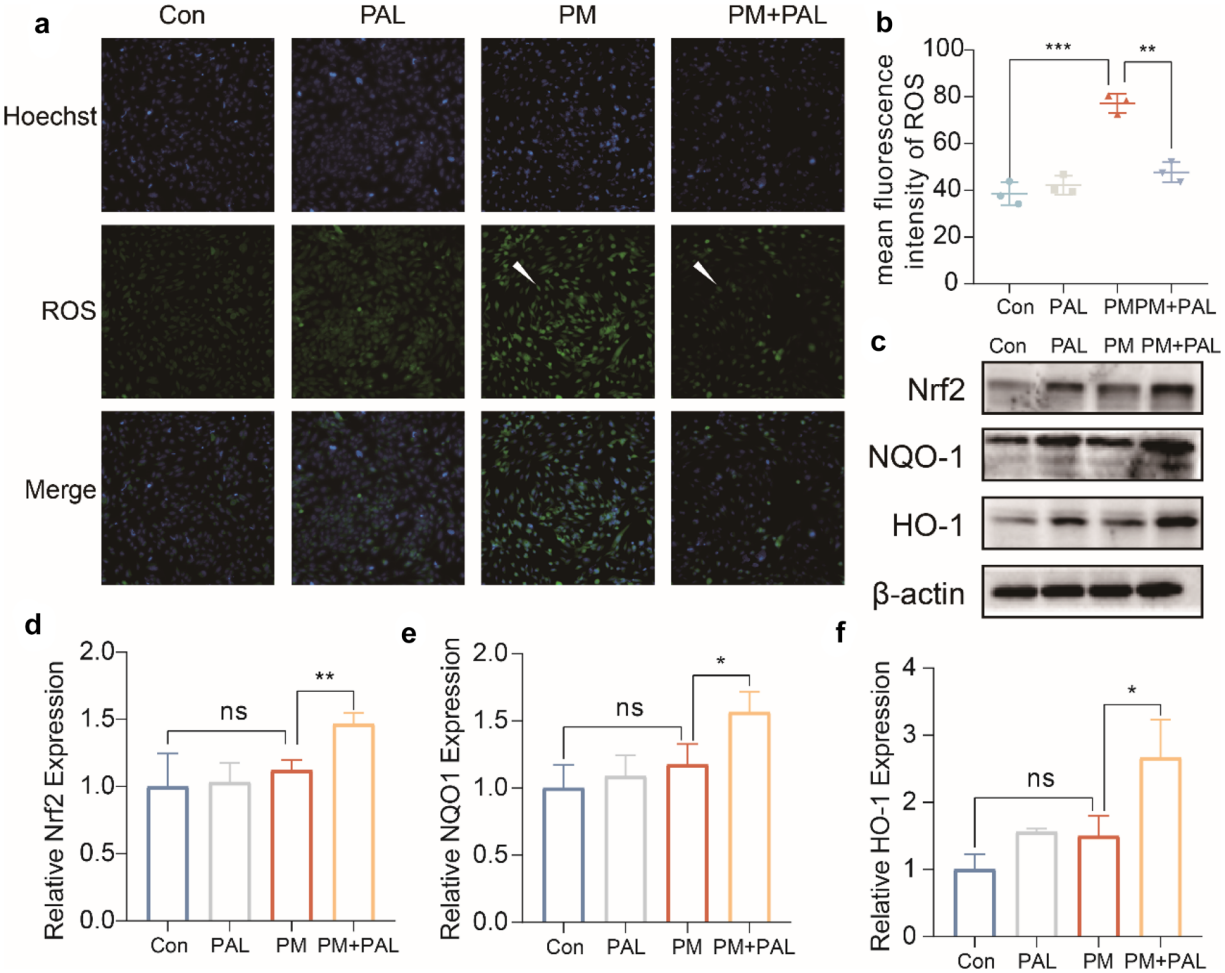
PAL alleviates the damage of Beas-2B cells induced by PM administration by enhancing the activation of Nrf2-related pathway. **a** Representative ROS staining of Beas-2B cells in different groups. The white arrows indicate the cells stained by ROS probe. **b** Mean fluorescence intensity (MFI) of ROS (*n* = 3). **c** Representative protein bands of Nrf2-related pathways by Western Blotting of Beas-2B cell in different groups. **d** Western Blotting analysis of Nrf2 protein (*n* = 3). **e** Western Blotting analysis of NQO1 protein (*n* = 3). **f** Western Blotting analysis of HO-1 protein (*n* = 3). *p < 0.05; **p < 0.01, ***p < 0.001. One-way ANOVA was used for multiple comparisons.

By TUNEL staining, we found that the proportion of apoptotic Beas-2B cells increased significantly after PM administration, while PAL could reduce the apoptotic proportion of Beas-2B cells (Fig. 4a, b). The protein of Beas-2B cells was further extracted for Western Blotting. The results of Western Blotting showed that PM administration could lead to the activation of NLRP3-related pyroptosis pathway, while PAL could significantly inhibit the activation of NLRP3-related pyroptosis pathway (Fig. 5c–i).

### PAL Significantly Activated Nrf2-Related Antioxidant Pathway in Beas-2B Cell Line

The results of ROS detection showed that the administration of PM could significantly increase ROS in Beas-2B cells, while PAL could significantly reduce the increase of ROS induced by PM (Fig. 5a, b). The results of Western Blotting showed that PM could significantly activate Nrf2-related pathways in Beas-2B cell line, while PAL could further enhance the activation of Nrf2-related pathways (Fig. 5c–f).

### PAL Significantly Inhibited the Activation of NLRP3-Related Pyroptosis Pathway by Activating Nrf2-Related Antioxidant Pathway in Beas-2B Cell Line

**Fig. 6:**
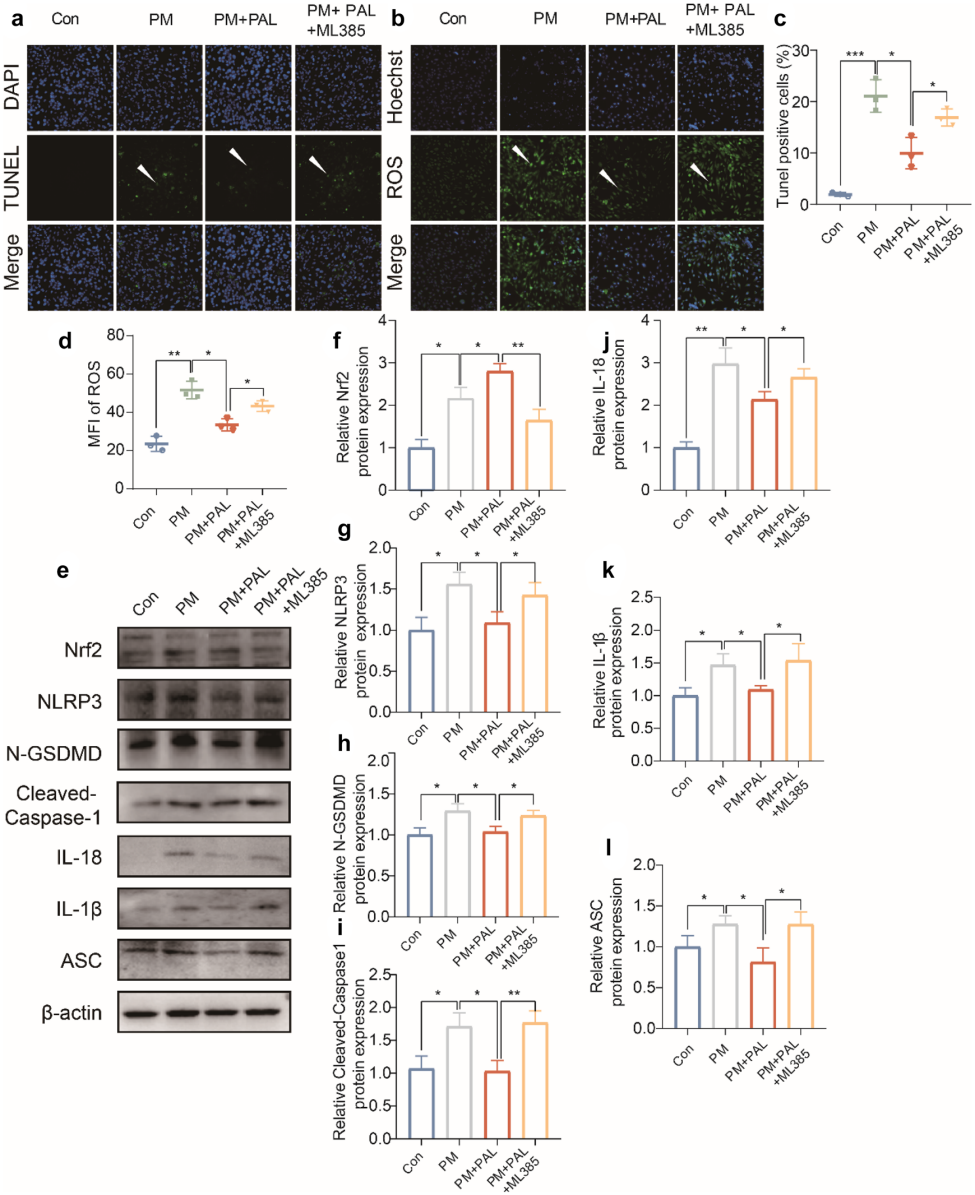
PAL inhibited the activation of NLRP3-related pyroptosis pathway by activating Nrf2-related antioxidant pathway in Beas-2B cell line. **a** Representative IF staining of TUNEL staining of Beas-2B cells in different groups. The white arrows indicate the apoptotic cells that we focus on. **b** Representative ROS staining of Beas-2B cells in different groups. The white arrows indicate the cells stained by ROS probe. **c** Percentage of TUNEL positive cell number relative to the total number of Hoechst (*n* = 3). **d** MFI of ROS (*n* = 3). **e** Representative protein bands of Nrf2 protein and NLRP3-related pathways by Western Blotting of Beas-2B cell in different groups. **f** Western Blotting analysis of Nrf2 protein (*n* = 3). **g** Western Blotting analysis of NLRP3 protein (*n* = 3). **h** Western Blotting analysis of N-GSDMD protein (*n* = 3). **i** Western Blotting analysis of Cleaved-Caspase3 protein (*n* = 3). **j** Western Blotting analysis of IL-18 protein (*n* = 3). **k** Western Blotting analysis of IL-1β protein (*n* = 3). **l** Western Blotting analysis of ASC protein (*n* = 3). *p < 0.05; **p < 0.01, ***p < 0.001. One-way ANOVA was used for multiple comparisons.

Through TUNEL assay and ROS detection, we found that increased PM-induced cell death and ROS could be inhibited by PAL; however, after ML385 administration, ROS levels increased and cell death numbers increased (Fig. 6a–d). The results of Western Blotting showed that administration of ML385 inhibited Nrf2-related pathway and led to activation of NLRP3-related pyroptosis pathway (Fig. 6e–l).

## Discussion

As an environmental pollutant, PM can induce various respiratory diseases by triggering acute inflammatory responses. In this study, it was observed that the inflammatory response caused by PM primarily targeted airway epithelial cells, which aligns with the findings of other researchers [21]. The development of novel treatments for PM-induced lung injury has been a focal point for researchers. Currently, extracts derived from diverse plant sources are increasingly utilized in the production or semi-synthesis of new medications. PAL is a prominent bioactive compound found in several plants including coptis, phellodendron, laminaria, barberry, and papaya. These plants have long been employed in traditional medicine practices in China, Korea, and India to alleviate various ailments such as jaundice, liver-related disorders, hypertension, inflammation, and dysentery [13]. In this study, we explored the effect of PAL on PM-induced lung injury, and the results showed that oral administration of PAL could reduce inflammation and have a significant protective effect on PM-induced lung airway epithelial cell injury.

After further investigating the mechanism through which PAL mitigates PM-induced lung injury, we discovered a significant reduction in NLRP3 protein expression within the pulmonary airway epithelium upon PAL administration. This finding suggests that PAL may safeguard airway epithelial cells by inhibiting the activation of the NLRP3-associated pyroptosis pathway. The results obtained from Western Blotting confirmed our hypothesis that oral administration of PAL effectively suppresses PM-induced activation of the NLRP3-related pyroptosis pathway in mice lungs. Numerous previous studies have demonstrated an association between PM exposure and increased pyroptosis in mouse airway epithelial cells [22]. Through our findings, it is evident that PAL can provide protection against acute lung injury induced by PM by attenuating the activation of the pyroptosis pathway in mice lung tissue.

When cells are damaged, they produce a substantial amount of reactive oxygen species (ROS), and failure to effectively eliminate excessive ROS will further exacerbate cellular damage. The Nrf2-related pathway plays a crucial role in combating oxidative stress, and its activation can significantly reduce intracellular ROS levels [23]. The results of immunofluorescence staining revealed that pulmonary airway epithelial cells exhibited an up-regulation of Nrf2 protein expression upon PM administration, with even greater increases observed after PAL administration. The results of Western blotting demonstrated that both lung tissue and Beas-2B cells displayed activation of the Nrf2-related pathway following PM stimulation, while PAL treatment further enhanced this activation. Malondialdehyde (MDA) is the final product of lipid peroxidation caused by free radicals *in vivo* and possesses cytotoxic properties. Superoxide dismutase (SOD) is an antioxidant enzyme that utilizes free radicals as substrates to neutralize them within the body [24]. We subsequently measured MDA concentration and SOD activity in lung bronchoalveolar lavage fluid (BALF) from mice, and the results revealed that PAL significantly attenuated the increase of MDA concentration induced by PM-induced lung injury while concurrently enhancing SOD activity to protect against lung injury. In cell experiments, stronger activation of the Nrf2 pathway more effectively cleared intracellular ROS and protected cells from damage. Previous studies have indicated that elevated ROS levels also contribute to the initiation of the NLRP3-related pyroptosis pathway [7, 8]. To investigate whether PAL inhibits this pathway by activating the Nrf2 pathway, we established a cell injury model using PM-treated Beas-2B cells as well as a treatment group receiving PAL supplementation along with an addition of ML385—an inhibitor targeting Nrf2—to assess its impact on pyroptosis. Our result suggested that PAL can clear intracellular ROS by activating the Nrf2-related pathway, thereby inhibiting the activation of the NLRP3-related pathway, which is consistent with the results of previous researchers [7, 8].

Many researchers have investigated the role of PAL in inflammation. Baoqi Yan *et al.* discovered that PAL suppresses LPS-induced inflammation in goat endometrial epithelial cells by inhibiting the TRIF-dependent NF-κB pathway [25]. Yunduan Song *et al.* found that PAL can reduce LPS-induced acute lung injury by interfering with the interaction of TAK1 and TAB1 [17]. These findings highlight the potential of PAL as a clinical intervention for inflammation. Our study revealed that PAL alleviated PM-induced lung inflammation in mice through activation of the Nrf2-associated antioxidant pathway and inhibition of the NLRP3-associated pyroptosis pathway, providing robust evidence for its therapeutic application in inflammatory conditions and offering a novel and effective approach to treating acute lung injury-induced by PM.

## Conclusion

By animal and cell experiments, we found that PAL, a natural compound derived from plants, mitigated PM-induced damage to the Beas-2B cell line and lung airway epithelium in mice. Mechanistically, we found that PAL alleviated PM-induced lung injury by promoting the activation of Nrf2-related antioxidant pathway and then inhibiting NLRP3-related pyroptosis pathway. PAL has the potential to be a new treatment option for PM-induced acute lung injury.

## Data Availability

All data generated or analyzed during this study are included in this article.
